# Thirty-Year Experience with Augmentation Rhinoplasty Using Silicone Implant: A Safer, Cheaper, Faster, and Effective Technique

**DOI:** 10.1093/asjof/ojaf018.017

**Published:** 2025-05-13

**Authors:** Albert Y Truong, Edmund K Kwan

**Affiliations:** Weill Cornell Medicine, New York, NY; Weill Cornell Medicine, New York

## Abstract

**Goals/Purpose:**

Many ethnic patients seeking rhinoplasty often present with insufficient dorsal projection, necessitating augmentation rhinoplasty. The debate over the optimal material for nasal augmentation continues, particularly between autologous tissue (bone, cartilage), allograft (cadaveric cartilage), and alloplastic implants (Gortex, silicone). Although autologous tissue is viewed as being safer with lower complication rates, it is susceptible to resorption, warping, aesthetic failure, and donor site deformity. Conversely, alloplastic materials may have higher risks of extrusion and infection. We propose that when performed correctly alloplastic augmentation may offer improved, consistent aesthetic outcomes with reduced operative time, lower morbidity, and a favorable complication risk profile.

**Methods/Technique:**

A retrospective review of patients who underwent silicone implant rhinoplasty by the senior surgeon from January 1995 to October 2024 was conducted. All patients underwent augmentation rhinoplasty with custom-carved silicone implant from grade 40 silicone block (Implantech) for the nasal dorsum. In addition to demographic data, surgical details such as form of anesthesia administered, type of graft employed, etc. and post-operative outcomes were recorded. Case details were also collected for any subsequent revision procedures.

In brief, the senior surgeon employs a closed rhinoplasty technique, which is described as follows. General anesthesia is administered, and 1% lidocaine with epinephrine is infiltrated into the entire nose. Bilateral intracartilagenous incisions are made. The cephalic portion of lower lateral cartilage is trimmed. A precise dorsal pocket, extending from the radix to the tip of the nose, is created utilizing both incisions in order to ensure that it is midline and subperiosteal. This step is critical to ensure optimal implant positioning and stabilization. The pocket is then irrigated with antibiotic solution prior to inserting a custom-carved, antibiotic-soaked silicone implant. The nasal implant, designed in an I-shape extending from the radix to the supratip area, is placed under no tension. Careful attention is paid to ensure that the tip of the implant is oriented midline and away from either intracartilagenous access incision sites. If additional tip projection is needed, cartilage is harvested from the septum or concha of the ear. The harvested cartilage is then shaped and inserted through the right rim incision into a subcutaneous pocket. All incisions are meticulously closed with 5-0 chromic sutures in simple interrupted fashion. A thermoplastic nasal splint is applied. All patients receive a 7-day course of postoperative antibiotics.

**Results/Complications:**

Over the span of the study, 1019 patients underwent augmentation rhinoplasty with silicone implant by the senior surgeon. A majority of the patients were ethnic, predominately East Asian females (90%) and aged 14 to 75 years. Closed primary rhinoplasty was performed in most cases while others required open techniques. Average operative time was 45 minutes. A tip graft was used in 764 cases (75%). The average follow-up period was 1 year (range between 1 month to 25 years). The secondary revision rate was about 10% (n=103), primarily due to unsatisfactory dorsal height (n=43, 4%) necessitating exchanges for different implants thicknesses. Malposition was noted in 4% (n=37) of cases, requiring either a new implant or reinsertion of the existing one. Infection occurred in 3 cases, which required removal of the implant. Extrusion rates were less than 1% (n = 9), most linked to secondary rhinoplasties. In contrast, L-shaped implants used by other practitioners showed significantly higher extrusion rates due to their larger dimensions resulting in increased tension on the nasal tip. In cases of extrusion among our I-shaped implant cohort, some were salvaged through removal and immediate reinsertion, while most were managed with removal under local anesthesia followed by secondary implantation three months later. 10 patients (1%) opted for explantation, primarily due to their desire to no longer have an implant, although most reported no significant issues. A majority of patients achieved satisfactory aesthetic results.

**Conclusion:**

Nasal augmentation is often necessary for patients seeking ethnic rhinoplasty. Silicone implants represent an ideal solution, particularly for patients lacking nasal dorsum and tip projection. When conducted by an experienced surgeon, the complication profile of implant rhinoplasty is comparable to that of other aesthetic facial implant procedures. Silicone implants for nasal augmentation carry a low, acceptable risk profile, are cost-effective, and offer predictable, satisfactory aesthetic outcomes.

